# Practical Essay on Beriberi, and Rheumatism

**Published:** 1838-01

**Authors:** 


### Art. VII.

*A Practical Essay on the History and Treatment of Beriberi, and Observations on some Forms of Rheumatism prevailing in India.* By Assistant-Surgeon John Grant Malcolmson, Madras Medical Establishment.—*Madras*, 1835. 8vo. pp. 343, 98.

To these essays was adjudged the prize offered by the Presidency of Fort St. George, of five hundred rupees, or of a gold medal of that value, for each of the best dissertations on the subjects mentioned in the title-page. They are described by the Medical Board of that Presidency as being not less remarkable for laborious research than for original and comprehensive views, and they have very properly been published by order of government.

I. Beriberi. We are conscious that the title of the first essay is little calculated to win the attention of the European reader; but, although its immediate subject is an endemic of regions so remote as Ceylon and the south-eastern coast of Hindostan, it is only necessary to state that the investigation of this disease has developed facts which have an important practical bearing on numerous intricate affections of the spinal cord, in order to ensure for Mr. Malcolmson's volume that attention from the profession in all countries to which its merits justly entitle it.

We shall not enter into the philological disquisition on the meaning of the word Beriberi. It is sufficient to state that this term is almost universally adopted by Europeans in India to designate a peculiar affection of the lower extremities, chest, and other parts, and which constitutes a very fatal endemic in that country. On the more important points of its prevalence and fatality, we learn that, of eighty-eight deaths which occurred among a body of native troops in 1827, fifty-two were owing to this disease, or five-eighths of the whole mortality, and five times more than the deaths from cholera or fever; and, during another period, whilst twenty deaths occurred from beriberi, there were but twelve from fever and four from dysentery. The deaths among cases *returned* as beriberi are one in four; but neither the total nor proportionate mortality is easily ascertained, an adherence to the names of Cullen's Nosology causing certain medical officers to designate the disease dropsy or rheumatism, and men who are discharged as cured occasionally dying in villages or garrison hospitals.

Though these circumstances tend to veil the real amount of fatality, enough is shown to prove the disease to be extremely destructive in the districts where it prevails; and Mr. Malcolmson applies himself with praiseworthy diligence to an enquiry into its causes. Like too many investigations of the same nature, however, this terminates unsatisfactorily, and the author candidly admits that, whilst he has demonstrated the inaccuracy of the causes assigned by preceding authorities, he is able to establish nothing in their room. The district in which the endemic chiefly prevails on the continent of India is about four hundred miles of the south-east coast of Hindostan, extending from Ganjam on the north to Masulipatam on the south. This tract is bounded landward by deeply-wooded hills, which are described as being the most destructive to health of any in India, except in the height of the hot weather. These hills approach sometimes within twenty miles of the shore, but in general are upwards of thirty or forty from it; and near Masulipatam, where the disease is comparatively mild, they are at a much greater distance. A description is given, and apparently a very accurate one, of the surface of this district, and likewise of its meteorology; and, from a consideration of all the circumstances, the author deduces the following corollaries:

"There certainly appears to be some connexion between the moisture of the surrounding country and the prevalence of beriberi; but much more complete and accurate series of observations are necessary to show to what extent this acts, in how far it is necessarily present, and by combination with what other influences it produces effects so different from those of similar or greater degrees of the same cause." (*Essay on Beriberi*, p. 37.)

——"Beriberi prevails in certain districts within forty miles of the sea, and at no great distance from mountainous forest tracts, and in which the rains commence early, are of considerable violence, and the face of the country much flooded, affecting chiefly adults and strangers from other districts and other parts of the same, who have resided from eight to twenty months in the place. This law will, I have no doubt, require to be greatly modified as our information is extended, but it will be useful at present in directing our enquiries." (*Ibid.*, p. 38.)

The influence of the ordinary causes of fever,—of sea-air and moisture,

—of the water employed as drink, and of the grain used as food, is passed under consideration in the enquiry, but without any decisive conclusion being attained. An intimation is given, however, that the deteriorated quality of the rice and other grains produced in this district may be instrumental in engendering the disease. The author quotes Professor Christison, to show that the diseases of grain which have given rise to epidemics in Europe have been caused by a damp, warm atmosphere, to which a low, moist soil and surrounding woods have contributed. Such influences are in operation in the places where the disease prevails, and, if taken in connexion with the injunction of native practitioners to patients labouring under beriberi to restrict themselves to a diet of wheat, (which is generally brought from the interior,) afford encouragement to the careful observation of the causes to which they seem to point. With respect to contagion, it is not generally supposed to be a cause, but attention is called to the circumstance that, in many instances, members of the same family have fallen victims to beriberi.

The following description will furnish the reader with a general idea of the disease, and certainly, by the place of precedence very correctly given to the paralytic symptoms, tends strongly to confirm the pathological views subsequently developed by the author.

"It usually commences gradually, with a feeling of numbness, sense of weight, and slight weakness and stiffness below the middle of the thighs, sometimes preceded by muscular pains. There is slight œdema of the feet and legs, especially along the tibiæ, often found to come on after the other symptoms. The walk is unsteady and tottering, even when the patient is not aware of weakness in the limbs, which are occasionally tremulous; spasms occur in the calves and soles of the feet, sometimes becoming general, and occasionally shooting to the chest and larynx, obstructing respiration and speech. The want of power often rapidly increases to almost total palsy, especially of the extensor muscles; and in a few cases the patient, after slight indisposition, suddenly loses the use of his legs. Rigidity and various painful affections of the nerves accompany the paralytic symptoms; and there is sometimes pain along the spine, commonly at the two last lumbar vertebræ. In some cases the disease goes no further, and a cure is effected; but more frequently the numbness extends upwards towards the abdomen, there is general sense of lassitude and aversion to motion, and the hands, arms, and chest (and in a few cases even the neck and lips,) are gradually benumbed. There is oppression and weight at præcordia, dyspnœa on slight exertion, diffused and irregular pulsation in the cardiac region, and the face and hands are puffy and œdematous. The patient is often found dead in bed, or sinks after several fainting fits or throbbings at the heart; or the œdema rapidly increases and extends up the trunk, violent dyspnœa and inability to lie down in bed comes on, with anxiety, cold sweats, cold extremities, rapid feeble pulse, urgent thirst, and partial suppression of urine. At the commencement, the urine is always scanty, of a deep red colour, without cloud or sediment, and possessing very peculiar properties; in some old cases, it becomes copious, turbid, and pale, with a large white deposit, and is passed with pain from an irritable bladder. The stomach is irritable in many bad cases, and pain and tenderness in the epigastrium is sometimes complained of; there is, in a few, pain in the abdomen, or a sense of heat is diffused over it and the chest. Effusion takes place into the chest, and more rarely into the abdomen; and there are now and then some signs of inflammation of the pleura or bronchi. In the early stage, the pulse may be full, hard, and frequent, or little altered: when the face is puffy, and there is weight and oppression at the præcordia, it is quick, often irregular, and usually small, although it is occasionally strong.

"Various dyspeptic symptoms occur; the bowels are often costive, the stools green and variously disordered, aud the eyes are often tinged yellow. The skin is rather cold, unless there is pyrexia, which is often present in the evening. The disease is sometimes fatal in a few hours, but is often chronic; and in these the patient is liable to sudden death, to rapid aggravation of the symptoms, or supervention of new and more formidable ones, by which he is soon carried off; and, if he survives these, he may live for a long time bedridden, dropsical, and a true paralytic." (P. 4.)

Mr. Malcolmson gives a detailed and elaborate exposition of the various symptoms, and, in the first place, of those of palsy. This portion of the work will be read with interest and instruction; our abstract of it must of necessity be so brief as to be calculated rather to lead the reader to consult the original, than to allay his curiosity. The author is of opinion that the lower portion of the spinal cord is the seat of the *primary* affection, and that this affection is indicated by an influence on both sets of nerves, sensitive and motory, derived from the *cauda equina*, and supplying the lower limbs. This morbid condition of the spinal cord has unquestionably a tendency to spread upwards, and, when the superior part of the body partakes of the disorder, the different sets of nerves are more unequally influenced; a difference for which we are furnished with the reasonable and ingenious explanation, that in the former instance the nerves are combined into bundles, in which those possessed of different functions must be in general equally affected by disease; and in the latter, where they branch off from distinct roots from the opposite surfaces of the cord, either may partake of diseased action without the other. The affection of the spinal cord, though always existing and essential to the disease, the author believes to be, in the first instance, rather functional than organic. Among the evidences of the accuracy of the pathological views here adopted, we should not omit to mention that, besides the imperfect or disordered action of the nerves of the lower extremities, for the source of which all sound physiology would lead us to look to the inferior portion of the spinal medulla, severe internal pain is felt in the sacro-lumbar region, which, it should be remarked, is not increased by pressure.

The affection of the nerves of sensation, indicative of the lesion of the spinal marrow, is depicted with great minuteness, and, we feel no doubt, fidelity. Numbness affects both sides of the body, but not always equally, and, in the early stages, very generally extends no higher than a little below the middle of the thighs, and, in slight cases, not above half-way up the legs, and, as the disease declines, successively descends to the ankle, instep, and toes; and, when the arms are affected, the skin is usually sensible over the upper two-thirds or half of the forearm, but, in either case, the numbness seldom terminates quite abruptly. The explanation, Mr. Malcolmson remarks, is, in the majority of cases, sufficiently evident by attention to the origin of the nerves and to the fact that, when the functions of a nerve are impaired by artificial pressure or disease, the extreme branches suffer first, and, as the obstruction increases, the larger trunks in succession; and that, on removing the pressure, the functions are restored in a reverse order. If the pressure is made on the cord itself, this is greatly modified by the origins of the different classes of nerves being unequally injured. The injury, for instance, of the roots of the ischiatic nerves, formed of the anterior sacral branches, will fully account for the loss of sensation of the legs; whilst the numerous branches of these nerves distributed on the soles of the feet about the roots of the toes, and on the calves of the legs, will explain the fact that these parts are more affected than almost any other; whilst the higher origin of the obturator, anterior crural, and cutaneous nerves explains the common termination of numbness one-third above the knee. In one case, the numbness was confined to the lower part of the *legs* and to the *hips*, which derive their cutaneous nerves from the sciatic or sacral plexus.

There is frequently diminished sensibility over the body, when the motory nerves are little affected. This the author believes to arise, not from the posterior column of the spinal marrow alone being injured, but from that general law of the nervous system, according to which the more delicate function of sensation is disordered by slighter causes than the motory. In many examples, however, the distinct nature of the two classes of symptoms is evident: sensation may continue unimpaired when the powers of motion are entirely lost; and instances occur in which entire loss of feeling has extended to the umbilicus, the nipples, and even to the neck, without palsy of the muscles of the trunk. So also, when the diseased action has extended to the upper part of the cord and the medulla oblongata, numbness of the lips, appearing to depend on an affection of the ganglionic branch of the fifth pair, is common in Ceylon, and is not unknown elsewhere; but loss of muscular power of the face has not been observed in beriberi.

Various modifications of sensation are observed in the affected parts, as in diseases of the spine in Europe, particularly pain and a sense of burning, especially of the feet, but occasionally occupying other portions of the body; a subject to which we shall have subsequently to revert. There are some interesting experiments and remarks on the influence of the disease over the actual temperature of the affected parts, for which our limits compel us to refer the reader to the original. On the affections of the muscular system, the author states that additional observations are required. Palsy is the most constantly present of all the muscular symptoms, generally coming on slowly, but now and then very rapidly. It exists in very different degrees, and, in one case reported by Dr. Herklots, amounted to total loss of power of almost every muscle of the body. In the majority of recent cases, spasmodic rigidity is also present; and in a few the flexor muscles were permanently contracted. The cramps are most distressing in the calves of the legs and soles of the feet; and, in one example, the muscles of the back were thrown into such rigid contraction as to give the appearance of opisthotonos.

The following are the proportions in which certain modifications of feeling and affections of the muscles occurred in sixty-five cases.

"Sixty had more or less paralysis, and in the other five it had been prevented by the early use of remedies; in one, almost the whole body was affected, and in some only a finger or toe; 57 had numbness of the feet or hands; in a few, a spot only on different parts of the body was affected, and in others the head and breast were the only places not benumbed; 48 had pain or soreness; 40 œdema; 33 spasms; 11 had the gait of the sheep; 12 tottering in walking; and 24 had sense of weight in limbs or thorax. There was a sense of coldness in the extremities in five; of biting of ants in three; in ten, sense of tingling; and in four, of the feet being covered with clay." (P. 86.)

Mr. Malcolmson proposes his views of the pathology of the disease with great but laudable diffidence, regarding it as a possible case that the membranes of the medulla are greatly, but not exclusively, involved in the morbid actions on which the symptoms depend. The brain, he remarks, rarely suffers, and the instances that occur do not often appear to be extensions of diseased actions from the spinal canal. When such suffering takes place, it arises from a febrile paroxysm, or from the state of the urinary organs; or it consists of effusion into the cerebral cavities, as a part of the general tendency to serous infiltration; but it is most frequently the effect of obstructed circulation from thoracic disease. From this exemption of the brain from immediate participation in the train of morbid actions, he deduces the inferences that they do not consist in inflammation of the substance of the cord, which usually extends to the brain, or of fluid within the arachnoid lining the dura mater, which would readily pass into the skull, the communication being free. The following is his account of the probable immediate seat of the primary affection.

"The pia mater of the cord (considered by some anatomists as a distinct membrane terminating at the foramen magnum,) is a strong resisting tissue, intimately adherent by its internal surface to the spinal marrow, and continuous laterally with the neurilema of the vertebral nerves and the ligamentum dentatum. The arachnoid membrane is nearly confounded with its external surface, from which it is reflected to cover the inside of the dura mater, to which it is very closely united, and it forms a cul de sac at the extremity of the canal. The dura mater is connected to the vertebræ by very loose cellular membrane, but is so intimately united to the margin of the foramen magnum as to limit the extension of disease upwards, while its morbid states are readily communicated to the other membranes. Its actions are usually slow, (Bichat *Traité des Membranes*, par Husson, 1816, p. 103, 167, &c.) Connecting these anatomical particulars with the symptoms, and with the fact of the disease more rarely extending to the brain than affections of the cord itself or of the arachnoid are stated to do, and with the severe pain experienced in some cases, in the situation of the cul de sac formed by the junction of the membranes, it may be safely proposed, as a probable inference, that the sheath of the cord partakes principally in the disorder; and that, through the continuity of the arachnoid, the morbid action is communicated to the proper membrane of the spinal marrow and nerves." (P. 96.)

These pathological views of Mr. Malcolmson are not left destitute of the support to be derived from the physical lesions discovered after death. The following description of the condition of the medulla in such cases we consider so confirmatory of the doctrine contained in the volume, that we request the reader's particular attention to it.

"The spine was opened from behind, and did not appear to contain any fluid, except what passed from without on opening it, and some slight infiltration of the cellular substance. There was no fluid external to the theca anteriorly, and the contents of the canal, at first view, from the second dorsal to the fourth lumbar vertebra, appeared healthy. At the fourth lumbar vertebra, to the left side posteriorly, there was a small mass of reddish coagulated lymph. This increased towards the sacrum, the cavity of which was nearly filled with a thick mass of firm lymph, evidently organized, of a reddish colour, with some fine vessels and longitudinal and transverse fibres. Towards the coccyx, it passed into the fatty substance. It was not confined to the posterior surface, but passed between the bone and sheath anteriorly, and some way along the sacral nerves, especially those of the left side. It was more firmly adherent to the lining of the canal than to the sheath of the nerves, which in several places appeared healthy below the lymph; in some others, the lining of the canal came away with it; and anteriorly the bone seemed softened, but this was very doubtful. The membranes and nerves did not appear otherwise diseased. Of these appearances the drawing gives a sufficiently accurate representation. Pursuing the dissection upwards from the second dorsal vertebra to the attachment of the sheath to the occiput anteriorly, a red mass, like a coagulum of blood, of considerable thickness and well defined, was found; which was probably the cellular membrane injected with a bloody fluid. It was confined to the anterior surface of the cord. On opening the theca, no fluid, congestion of the vessels, or disease of the arachnoid, or of the proper membrane or substance of the cord, could be detected. The nerves supplying the lower extremities, and some of the abdominal twigs without the spine, were healthy.—Head: Much bloody serum escapes from the scalp; a very little fluid between the arachnoid and pia mater; convolutions well marked, and the substance of the brain is firm and healthy; a very little water in lateral, none in third, and a little in fourth ventricle. No fluid at the base of the brain, and the cerebellum is healthy." (P. 110.)

This case is illustrated in the original by an engraving.

It is remarked that there is much ambiguity in the inferences to be drawn from serous effusion between the dura mater and inner membrane of the cord, on account of the free communication which this space has with the cavity of the cranium; but Abercrombie has observed, that this is removed when the fluid in the spine is bloody, while that under the arachnoid of the brain is colourless. These circumstances occurred in the following dissection.

"There was slight serous effusion between the dura and pia mater, without signs of diseased brain. On examining the spinal marrow, there were redness and vascularity of its investing membranes along their whole extent, and slight effusion of reddish coloured serum, with general vascularity of the substance of the cord in the lumbar region." (P. 116.)

Mr. Malcolmson very properly regards mere vascularity within the spinal canal as a very deceptive appearance. He is of opinion, too, that cases will occur in which alterations of the cord may be so minute as to elude our search, even when existing; and in purely functional cases we cannot, of course, expect to meet them.

Having concluded his remarks on the direct effect of the spinal disease, the author proceeds to consider those affections of distant organs so often associated with it, but which, none of them being universally present, cannot be considered as essential. He takes first in order the morbid changes in the urinary organs and secretion, which appear to have an important influence in the production of the other derangements.

In recent cases, the urine is scanty, of a deep red colour and pellucid. In some cases it is suppressed; but very scanty secretion is the more usual condition, even in the worst cases. There is a difficulty in discovering whether the change in the urine precedes or follows the spinal affection; but the probability is, that the renal disorder is secondary. In Mr. Malcolmson's experiments, the urine possessed powerful acid properties, was of low specific gravity, and was deficient in urea, without generally containing albumen, or, if any was present in any case, it was in insignificant quantity. The acidity was found to be owing to the presence of free phosphoric acid; as, from 1000 parts of urine, 11 grains of phosphate of lime were precipitated by the addition of pure limewater, giving, by the table of equivalents, 5.5 grains of phosphoric acid.

"When the free acid was almost neutralized, the colour of the precipitate changed to a most beautiful pink; purpuric acid appearing to be developed by the limewater precipitating its coloured salt, after precipitation of the free phosphoric acid, which can be conceived only to take place as a free acid of less affinity. The deep red of the urine is not diminished by the separation of these substances, and is destroyed by nitric acid and boiling, and is altered by sulphuric acid. The urine, kept for two weeks, retained its acidity and red colour; and its smell, though disagreeable, was not urinous or ammoniacal." (P. 136.)

It was remarked that, in rapid cures, effected by medicines which act principally on the nervous system, the urine speedily regained its healthy appearance.

In cases which remain long unrelieved, or which have frequently relapsed, the urine passes into the condition in which it is found in ordinary paraplegia, or injury of the lower part of the spine. It is then copious, sometimes as much as eighty or ninety ounces in twenty-four hours; generally pale in colour, although sometimes reddish, of a specific gravity from 1.020 to 1.025, uncoagulable by heat, and exhaling a strong ammoniacal odour without fetor. This state, which Dr. Prout considers as indicating what he calls the phosphatic diathesis, has been rendered so familiar to the British reader by the labours of this distinguished chemist, and by the melancholy experience furnished by injuries of the spine, that our extracts from this portion of the work will consist less of a general analysis than of any facts or opinions that appear to us to belong especially to the writer. This we mention by way of explaining a degree of abruptness of transition from one subject to another in our brief extracts.

Coinciding with the phosphatic condition of the urine, the author has observed a discharge of what he considered to be ammoniaco-magnesian phosphate from the bowels; and in another case he found the spleen studded externally with calcareous tubercles, and a few superficial tubercles of the same character in the lungs. The secretion of urine is not uniformly copious, but is occasionally greatly diminished; and the consequence has been anasarca, unattended by inflammatory symptoms, and not difficult to remove; and, in a case of this kind, the author would dissent from a precept of Dr. Prout, generally valid, that, in the phosphatic diathesis, we should abstain from diuretics. He regards, in common with all who have witnessed its sequel, this condition of the urine as indicative of great danger; and considers the risk of its occurrence a reason for deviating from the cautious plans inculcated by certain writers on Palsy, of waiting till all chance of excitement is over, before resorting to peculiar stimulants useful in diseases of the nerves; a view in which we concur with him, though admitting that great experience and discrimination are required for its practical application.

The indications of structural change in the urinary organs attending this alteration of secretion are briefly but clearly portrayed. There is weakness in the loins and pain in the region of the kidney, which in one case was observed to occur previous to the earthy deposition, when the change from the acid to the alkaline state of the urine was about to take place. The ureters, in one case, could be felt thickened and tender through the abdominal muscles during life. When the bladder is distended, there is great tenderness in the hypogastrium, increased on pressure; and, when the disease has made much progress, there is fulness and sense of weight in the pubis, the bladder can be felt thickened, and may be pressed between the finger and thumb. These organic changes render the hope of cure very slight; and, as an illustration, we may state that, of nine cases of this description, Mr. Malcolmson found six fatal, two were lost sight of, and but one was known to be cured. The condition of the kidneys and bladder, as displayed by dissection, is shown to be essentially the same in beriberi and sporadic disease of the spine, by the comparison of cases of each form of disease. An opinion of Dr. Alison's is controverted, that the kidney is affected in consequence of the extension of disease from the bladder. Mr. Malcolmson, on the other hand, thinks that the latter organ suffers partly from the irritating secretion, and also from a similar irritation to that affecting the kidneys, probably communicated along the numerous branches from the anterior sacral and lumbar nerves. That the kidney is previously affected appears from the pain early experienced in its site, and from the facts that lime and acid may be both simultaneously in excess in the urine; and that ammonia, either naturally produced or added, causes the phosphatic deposit only when the phosphatic change in its qualities had previously taken place. The nearly total absence of mucus in the deposit in the early stage also shows that the secretion of the bladder is not, as supposed by Dr. Alison, first increased or altered; and the products of its decomposition are evidently very different from the almost pure carbonate of ammonia extricated from the urine immediately on its being passed. An opinion of Dr. Scudamore's is likewise corrected. This writer supposes that the white deposit is prevented from crystallizing by the admixture of mucus. Mr. Malcolmson remarks, that the phosphate of lime is seldom anything but pulverulent, the crystallized phosphate being the ammoniaco-magnesian compound. The opinion of Brande, that phosphate of lime calculus is formed only in the bladder, is refuted by the plain fact that, in Case ii. of the Essay on Beriberi, it was found in the pelvis of the kidney. The author's experience of the condition of the urine in beriberi enables him to confirm the doctrine of Prout, that the fusible sediments are deposited in the early stage of the phosphatic diathesis; and, as the symptoms become more severe, the proportion of phosphate of lime to the ammoniaco-magnesian phosphate gradually increases.

Berzelius* and others, observing deposition of the phosphates to be followed by paraplegia, consider the altered action of the kidney as the cause of the loss of power in the lower part of the body. Dr. Prout, too, has remarked that various distressing sensations in the limbs, and even palsy, have been produced by the thickened bladder pressing on the nerves.† In such cases, Mr. Malcolmson would reverse the order of causation, and view the altered secretion and structure of the urinary organs as induced by a spinal affection, which, in its early stage, from the insidious nature of the primary symptoms, has been overlooked. We think this infinitely the more probable and rational view, though we might hesitate before assenting to the proposition "that in *all cases* the phosphatic diathesis arises from affection of the spinal cord." Mr. Malcolmson's experience in beriberi, however, is, so far as it goes, strongly corroborative of its truth.

* See Medico-Chirurgical Transactions, vol. iii.

† Diseases of the Urinary Organs, p. 253.

The œdema, so important a part of the disease, bears a proportion to the affection of the nerves and the condition of the urinary secretion. In its simplest form it is confined to the lower extremities, occupying a space proportionate to the extent of the numbness, pain, or irregular contractions. In acute cases and when the quantity of urine is much diminished "its progress upwards is exceedingly rapid, and it frequently ascends higher than there is any marked numbness, and as it rises up the chest, water is too frequently effused into the cellular structure of the lungs and rapidly destroys the patient." This œdema of the lungs is a frequent cause of sudden death in the disease; and, as its importance justifies, it receives copious illustration from numerous and well-reported cases. Besides the deficiency of urinary secretion, other causes, especially affection of the heart, and dropsy of the pericardium, conspire to produce it, and it often supervenes very suddenly and unexpectedly when the patient is thought convalescent.

Besides the lungs, the face furnishes an exception to the general law of this disease, that the dropsy is commensurate in extent with the numbness; for it is frequently œdematous when the ganglionic branches of the fifth pair are not affected. Any pectoral disease, as is familiarly known, will cause puffiness of the face, but its actual œdema is with few exceptions dependent on affection of the heart or its envelopes, and especially on dropsy of the pericardium. Our own experience, and we believe that of almost every practitioner who has made cardiac diseases an object of attention, might be adduced in confirmation of the author's opinion. In beriberi this swelling of the face is sometimes the first symptom of dangerous thoracic disease, and, where it is present in the slightest degree, there is the greatest risk of sudden death. This subject, like every other in the volume, is illustrated by valuable cases and dissections, and in the majority of the latter, besides effusion into the pericardium, a fluid state of the blood is observed. The author mentions this condition of the blood as a frequent but not a universal occurrence; and very properly points out the facility which it affords to cadaveric congestions, and the errors into which it might lead the inexperienced in their conclusions respecting morbid appearances.

Whilst the author regards diminished nervous power through its effect on the capillaries as the principal cause of the anasarca, he does not overlook the influence of other agencies in its production. Among these he concurs with Dr. Christison and much earlier writers in classing diminished urinary secretion, though he thinks its effect less than that of the diminished nervous power, the œdema sometimes not being removed by a copious flow of urine of the natural quality, whilst in other instances it is corrected by the treatment which has restored the functions of the nerves, though the urine continues to be scanty and of morbid quality. The form of anasarca which follows exhausting diseases, and which is truly a disease of debility, is not uncommon in beriberi: the legs are much swollen and the emaciation great. It often attacks those who have undergone severe mercurial treatment.

We have already adverted to hydrops pericardii, and from the chapter on the cardiac symptoms it will be found, that, besides this important affection, pericarditis, hypertrophy, and passive dilatation of the heart, and degeneration of its muscular fibre are also associated with beriberi and are often the cause of its sudden fatality. We pass rapidly over this chapter, because, though it comprises an elaborate description of the symptoms of these affections required to establish the fact that they frequently form a portion of this singular endemic, we do not perceive that it contains much information regarding them likely in the present state of our knowledge to be considered valuable in Europe, beyond the fact of their connexion with beriberi, and the influence this may have on our views respecting the etiology of such disorders occurring under different circumstances. The foundation of those connected with beriberi, Mr. Malcolmson discovers in the doctrine now recognized in pathology of the influence of the nerves on the sanguiferous system, "the fact that continued nervous irritation always tends to produce inflammatory action."

"It is exceedingly probable, (he says,) that the pericardium suffers in this way, the symptoms of cardiac affection seldom appearing when there are not unequivocal symptoms of the part of the spinal cord, above the origin of the nerves which assist in forming the cardiac plexuses being more or less diseased, and in the same patient it may frequently be observed that the lower part of the body may suffer much, and recover from several relapses, but on a return of the disease, in which symptoms of the affection of the spine having extended upwards are apparent, effusion or irritation of the cardiac region carries off the patient. We shall also find that treatment which cannot be supposed to have any sudden effect, except through the nervous system, removes the cardiac affection in a very short period." . . . "The nature of the part exerts a certain influence in producing the diseased actions in question. It (the pericardium) is a compound fibro-serous membrane, and enters readily into the same train of morbid action as other tissues of this class, or of either of those of which it is compounded. If, then, we are right in considering, that the fibro-serous envelopes of the spinal marrow are diseased, it will not appear improbable that similar actions will be readily excited in the pericardium."

The author illustrates this latter view very appropriately by the analogy of rheumatism. In one point we cannot agree with him. He assigns the imperfection of the union between the fibrous and serous portions of the compound membranes, and the feebleness of the sympathies between them, as reasons why pericarditis and many of the complicated phenomena of rheumatism and beriberi are nearly unknown in childhood. Now, at this period of life, pericarditis and ultimately hypertrophy or other disease of the heart itself, associated with rheumatism, is by no means rare in proportion to the number of such subjects attacked with this disease. We have, on the contrary, been very forcibly struck with the number of persons "in early youth," who are affected with rheumatic disease of the heart.

As might be expected, other organs, besides those we have enumerated, became the seat of numerous secondary affections of this multiform disease. Of these not the least interesting are the affections of the larynx. These are strikingly corroborative of the views of Dr. Ley in his essay on Laryngismus Stridulus, though from the dates of their respective works, it is impossible that the doctrine of Dr. Ley should have been known to our author, whilst it is highly improbable that the former writer should have seen the Essay on Beriberi. Two distinct modifications of laryngeal symptoms are observed in the complaint, aphonia without difficulty of breathing, and spasmodic constriction of the glottis threatening instant suffocation. The branches of the eighth pair are naturally referred to as the source of these symptoms, the aphonia without difficulty of breathing being ascribed to the condition of the recurrents, the branches of which are distributed to the crico-arytenoideus and thyro-arytenoideus, whose office is to expand the glottis; whilst in spasmodic constriction of this aperture, the superior laryngeal branches are supposed to be affected. If we mistake not Dr. Ley's opinion is that, in the latter case, the paralysed condition of the recurrents contributes to the constriction of the glottis by the withdrawing of the antagonizing power to the influence of the superior laryngeals. But this difference diminishes only slightly the coincidence in sentiment; in each case the superior laryngeals being the supposed active cause of the constriction. It should be borne in mind, moreover, that although the manifest morbid condition, closure of the glottis, exists alike in both diseases, the cause in laryngismus stridulus is pressure on the recurrents from tumefied glands, and in beriberi affection within the spinal canal.

The author refers the abdominal symptoms to the influence of the disease on the eighth pair and sympathetic, finding that when the lumbar nerves are so much injured as even to paralyse the abdominal muscles, there is but little affection of the stomach. The intimate connexion of the eighth pair, through its branches joining the lower cardiac plexus, will lead us to expect that the parts directly supplied by the nerve, will correspond in its morbid actions with the pectoral affections; and similar actions will in many cases be extended through the solar plexus, and the direct inosculations of the sympathetic with the lumbar nerves, to other organs. The stomach is sometimes affected with inflammation, as likewise is the peritoneum of the intestines and general cavity of the abdomen. Of ten dissections there were traces of inflammation with abdominal effusion in five, effusion without inflammation in one, inflammation without effusion in another, and in three there was no abdominal affection. "The bowels are usually slow, and the evacuations are frequently composed of disordered bile, sometimes greenish, at others black, like clay, white or lumpy. The liver frequently suffers, but seldom with severe symptoms. Those most usual are, yellowness of the eye, sallow complexion, and fæces displaying a superabundance of bile.

Referring those who have a prospect of being called on to combat this disease to the work itself, for some valuable remarks on diagnosis and prognosis, we pass to the subject of treatment. After remarking, with much truth, that general principles when applied to unknown complaints are worse than other kinds of empiricism, he proceeds to consider the different remedies employed in beriberi, and in the first place bloodletting. We scarcely need remark that this remedy is not recommended indiscriminately, but according to circumstances. In inflammatory affections of the heart and pericardium, great benefit is, as might be presumed, derived from it; and even in more obscure cases, where inflammation is latent, excepting so far as it is indicated by a scanty secretion of high-coloured urine with deficiency of urea, should the pulse be firm, it may likewise be advantageously employed. In affections of the pericardium where water exists within the unyielding membrane, and restrains the action of the heart, thus producing smallness and weakness of the pulse, bloodletting is well-supported and produces at least temporary relief. Cupping and leeches over the cardiac region are likewise very beneficial.

The author has not found bleeding required for hepatic symptoms; but local bleeding produces benefit in the inflammatory affections of the stomach, kidneys, and bladder. Both general and local bleeding are beneficial when there is much pain of the spine; but the spasmodic symptoms, whether situated in the legs, larynx, or elsewhere, are not relieved by bleeding of any kind.

There are three classes of cases in which bleeding has been uniformly fatal: the first is œdema of the lung which has taken place suddenly, a dangerous condition under any treatment; but by the use of stimulants and antispasmodics, it may for a time be surmounted, though apt to recur in an aggravated form. The next is spasmodic dyspnœa, accompanied with lowness of pulse, cold extremities, and prostration of strength. For this condition, stimulants and warm frictions to the limbs have appeared to be very beneficial. In another class, in which there is a spasmodic, enfeebled, or obstructed action of the heart, with paroxysmal dyspnœa, cold sweats and tendency to syncope, arising from various causes, but in some instances from water in the pericardium with softening of the heart, bloodletting is likewise fatal, whilst purgatives produce pernicious effects. Stimulants, with large doses of laudanum, sulphuric ether, and warm frictions, are the appropriate remedies.

*Mercury*, the author remarks, has in a few instances aided bloodletting in subduing the inflammation of the membranes. There are, however, exceptions, and his general opinion is adverse to the mineral, as he considers that it possesses no salutary influence over the nervous symptoms, and does not prevent the accession or the return of the visceral affections. The disease has attacked patients when under the influence of mercury for other complaints, and this medicine has been found to aggravate the paralytic symptoms. *Purgatives* are in general very beneficial, and of these Mr. Malcolmson considers compound powder of jalap the best; one or two doses of this medicine, he remarks, frequently removing the œdema of the lower extremities; but when the disease is confirmed, and the phosphatic diathesis prevails, the disordered secretions of the bowels are best evacuated by mild medicines. *Diuretics* are of importance in the removal of the œdema and in preventing its extension upwards; but their utility seems limited to these objects, for they are destitute of any influence over the spinal affection. Squill with *small* quantities of blue pill or calomel, so that the mouth may not be affected, given conjointly with tincture of digitalis and nitrous ether, is a suitable medicine, so likewise is the acetate of potash; but our author thinks cream of tartar the best of the class.

Under certain circumstances, internal stimulants are of use, but we must refer the reader to the original for the specification of these circumstances. Of those employed the root of the moringa tree (Hyperanthera Moringa) and preparations of iron, such as the tincture of muriated iron, are spoken of with most approbation. Frictions of the body and limbs with stimulating liniments are recommended, and of the substances employed for this purpose, turpentine, ammonia, and cajaput-oil, have been found most effectual. Blisters, sinapisms, ointment of tartarized antimony, caustic issues, moxas, setons, stimulating frictions to the spine, have all been used. Of blisters and issues he speaks more favorably than of the other means of counter-irritation. Walking, or immersion of the feet in hot sand at noon is a favorite remedy of the natives, and receives that attention from Mr. Malcolmson which native practices very generally and properly do from our medical officers in India. An hospital for convalescents was formed at Chicacole where this practice was adopted, and, conjointly with the change of climate the inmates were enjoying, it seems to have been very beneficial. Benefit has been derived in a few instances from galvanism transmitted through the spine. Nux vomica did not produce any useful result; and strychnia does not appear to have been tried—perhaps for want of a supply of the medicine.

*Treeak Farook*, an expression compounded of a corruption of the Greek θηριακὰ and of an Arabic word which may be translated "optima, or præstantissima," is the name of a remedy of the disease popular among the natives of India. Mr. Malcolmson had some difficulty in learning what this remedy was; but finally discovered that it was the old and very compound medicine, the Theriaca Andromachi, prepared at Venice. It has the power of producing rapid absorption of the effused fluid, and in the opinion of many persons, including our author, its beneficial influence over the palsy has been clearly ascertained, the numbness and peculiar unsteady walk of the patient are diminished by it. Over the affection of the pericardium, and œdema of the face and hands depending on this affection, it has a more prompt influence than over the œdema and numbness of the lower extremities. A smart purge should precede the employment of this medicine, and purgatives are frequently necessary in the course of the treatment. Cream of tartar, too, is sometimes necessary to aid in removing the œdema. The *treeak* has been found most useful in that form of the disease in which there is little excitement, the pulse being usually, but not always, feeble and small. Its virtues seem sometimes to be counteracted by inflammatory affection, but the author does not feel himself able to state the extent to which this counteraction operates. Mr. Geddes, an authority evidently much esteemed by Mr. Malcolmson, ascribes to it the power of lowering the pulse. This is disputed as a general fact; when the pulse was frequent from constitutional irritation of the whole system, no change was caused by the medicine, but when from cardiac affection, especially dropsy of the pericardium, it was restored rapidly to a natural state. It is evidently a medicine of great power over many symptoms. The form of its preparation is a very complex one. In a general view it may be said to consist of many stimulants and tonics, with opium. It seems probable that our Indian practitioners will discover which are the essential ingredients of this singular compound, and accomplish the same amount of good in a less costly and operose manner. We think the subject deserves further investigation, both in the direction we have adverted to, and with reference to the influence which this *farrago*, or its essential ingredients, may have over paralytic and cardiac affections in general, as well as those which constitute a portion of this very singular disease; and, should he have the opportunity, we know no one more likely than Mr. Malcolmson to be successful in such a research. Another medicine, introduced into European practice from that of the natives by Dr. Herklots, received from this gentleman the name of *oleum nigrum*: it is an empyreumatic oil obtained by a rude process of distillation from the seeds of the Celastrus nutans combined with a small proportion of benzoin, cloves, nutmegs, and mace. Mr. Malcolmson thinks this medicine has less power over the œdema and perhaps more over the nervous affections than the treeak farook, and it is through the latter effect only that it removes the hydropic symptoms.

II. Rheumatism. (*a.*) *Rheumatism in Natives.* This is described as a very frequent disease, and one which, though not often fatal, is the cause of more men being lost to the military service than all other diseases put together. In some instances it arises from direct exposure to cold and wet, and in character approaches to the disease observed in colder climates, requiring a cautious employment of a treatment similar to that applied to Europeans. In the majority of instances, however, it is a low, chronic form of disease, with little heat in the joints, but little amenable to medicine, and laying the foundation of numerous constitutional ailments. The author gives a very precise and copious enumeration of the parts affected, concluding with the remark that the tissues most generally involved in the disease are the ligamentous and tendinous, the periosteum, and more rarely the muscular, and many others are occasionally engaged. There may be no constitutional affection at the commencement, but, in a large proportion of protracted cases, the patient loses flesh and strength, and a cachectic habit is the consequence.

The indications of this cachectic condition are, loss of strength, emaciation, stooping, languor in motion, pale or puffy countenance, despondency and indolence. The abdomen is puffy or tympanitic, the digestion depraved, the appetite bad, the bowels are costive, and the evacuations dark. The tongue is generally swollen and white. In aggravated cases there is a burning sensation in the abdomen, which is extremely distressing, and probably depends on chronic inflammation of the mucous membranes. This affection proves frequently fatal with symptoms of diarrhœa and dysentery.

The remedies of this *cold* variety of rheumatism, (to use Mr. Malcolmson's expression,) are stimulating frictions to the affected parts, flannels and other warm clothing, blisters, warm baths, Dover's powder, guaiacum, and decoction of the woods. Of colchicum the author speaks slightingly, but supposes that its failure may arise from the decay in virtue of the acetous solution with which the military hospitals are supplied. From the reputation enjoyed by a hot sulphurous spring in the bed of the Godavery, and from the remarks of Dr. Bardesley, he is disposed to augur favorably of the effect of warm sulphur-baths on the disease. Of all its remedies, however, mercury is the chief, excepting in cases where pain, numbness, or pricking in the calves of the legs, arising from beriberi or neuralgia co-exist with the disorder, or where there were strumous ulcers or swellings. Even when pains and swellings of the bones and joints could be distinctly traced to mercurial action, or exposure when under such influence, still was the beneficial effect of the mineral marked and sudden. One grain and a half of calomel two or three times a day, combined with three grains of antimonial or James's powder is the mode recommended for administering the medicine.

(*b.*) *Rheumatism in Europeans.* This is the subject less of a formal treatise than of illustrations of important and interesting points in its history. The common acute rheumatism of Europe, as has been already remarked, is seldom seen in India, and when cases in some degree resembling it occur, they are not attended with that full development of local symptoms and fever, and with the buffy blood observed at home. Among the causes of the disease in general, cold acting on weakly constitutions is a frequent one, though numerous cases, which cannot be traced to any cause, occur during hot weather. In feverish districts rheumatism is very frequent, partly from the moisture and variableness of the climate, but often from this disease originating in long continued attacks of intermittent and remittent fever. The rheumatism arising in this way manifests itself in neuralgic pains and inflammation of the periosteum. The author controverts the supposition which might naturally arise in such cases of some venereal disease existing. He finds nodes a common symptom of rheumatism consequent on protracted fevers, and likewise on that which results from the use of mercury. He states from extensive experience, that pains in the limbs, periostitis, and nodes, arise in India from mercurial action for whatever reason it may have been induced. On this ground he applies himself to the correction of an error, into which Dr. Alison had been in some way led, when he stated it as "an ascertained fact" that in tropical climates, symptoms resembling syphilis never result from the use of mercury. Mr. Malcolmson has observed various affections of the skin arise from its use, especially an eruption of inflamed pustules over the body and face; and in one man who had not had any venereal complaint for twelve years, repeated courses of mercury for hepatic affections and fever, and subsequent exposure on a march in the wet season, caused severe nocturnal pains, tumefaction of the tibia, an eruption of round dark-coloured scaly blotches over the body, and very slow ulceration of the throat, of which he died. The author says that rheumatic pains follow immediately the use of mercury, at a time when rheumatism from other causes is almost unknown. The nature of the disease for which it is administered, does not seem to make much difference in the frequency and severity of the pains, as they follow hepatitis, dysentery, and simple membranous inflammation when treated by mercury.

Certain visceral affections are described by the author as the sequel of chronic rheumatism. White diarrhœa is a common symptom in these supervening diseases. Enlargement of the liver, inflammation, ulceration or abrasion of the mucous lining of the intestines, a tubercular condition of the peritoneum, and effusion into the abdominal cavity, are the changes, as we gather from the hospital reports, found in fatal cases.

(*c.*) *Burning of the Feet.* A considerable portion of the work is dedicated to the consideration of a singular complaint, only observed since the Burmese war, and called from its most urgent symptom "burning of the feet." Some confusion has arisen between this disease and beriberi, of which burning of the feet is an occasional symptom. Only by extensive observation could the question be cleared up; and this Mr. Malcolmson was enabled to effect by observing that on the return of the troops from Ava, in 1826, the corps sent to the northern division were suffering from burning of the feet; but no beriberi showed itself till some months after, when the other complaint had disappeared, and the period of residence and the season exposed them to the endemic of the country.

He considers it a disease totally distinct from beriberi and from rheumatism.

The sensation whence the disease derives its name extends over the surface of the lower extremities which are affected with severe pains, in many cases occupying especially the soles of the feet and calves. In some cases the burning has extended to the hands, and in a few to the whole body and even to the face. Besides the pains and emaciation, symptoms of a generally depressed habit are present. The skin is dry and harsh, often scaly or covered with itch; there is irregular fever; debility; and a tongue, pale, swollen, smooth, or furred, or red when the intestinal mucous membrane is excited. The gums are occasionally swollen and soft, and there is night blindness as in some forms of scurvy. Cough and dyspnœa are not uncommon symptoms, the digestion is in almost every instance impaired. The abdomen is often tympanitic and tender to the touch; diarrhœa, dysentery, pain in the course of the colon or around the umbilicus, frequently occur, and increase the emaciation, or destroy the patient. Dropsical swellings of the legs are common; they are usually the result of debility alone, and disappear on the patient's preserving the recumbent posture; but they are sometimes the effect of organic changes, and complicated with effusion into the cavities. Men labouring under the disease are subject to sloughing ulcers.

The lesions discovered on dissection are of a complicated nature. The lungs are often tuberculated, and fluid is frequently accumulated in considerable quantity in the pleural and pericardial cavities. In the abdomen there is generally observed serous effusion, a thickened and opaque condition of the peritoneum, vascularity and ulceration of the intestines, particularly of the ileum and colon. Fluid is sometimes found in the ventricles of the brain, and sometimes within, and very generally and abundantly around the spinal theca. In some cases softening of the cord has been observed.

The remedies recommended for burning of the feet, are nutritious food and removal from the damp climate in which the disease is engendered; and the author is very urgent that these measures should be adopted before irremediable organic changes occur. He states that he has not seen any disease in Europeans of the same nature as this; but refers to a case of neuralgia in the sciatic nerves and spasms of the legs and thighs, recorded in the London Medical and Surgical Journal for April 1832, as bearing some analogy to it. We cannot vouch for the resemblance between all the parts of two diseases, one only of which we have seen; but the most uniform and distressing symptom of the disease described by the author, the pain and sense of burning in the feet and legs, is a very common accompaniment in this country, especially in females, of those complicated organic changes which indulgence in ardent spirits engenders. So frequently have we observed this affection in female tavern-keepers, that among our professional brethren, merely to avoid circumlocution, we have been in the habit of calling it the public-house disease. The most evident organic changes with which these sensations have been found associated were seated in the liver, stomach, and intestines. The disease we have found almost uniformly fatal. Circumstances, which will be readily understood, have prevented our discovering the condition of the spinal medulla after death; and we have been obliged to rest satisfied with referring the sensation in the feet and legs to that principle designated by Dr. Marshall Hall "the reflex function of the spinal cord," and suppose that a perception of the diseased condition within the abdomen existed in the nervous centre, and that sympathetic sensation was experienced in the extreme branches of the sciatic nerves.

We thought we should best consult the interests of the reader by presenting him with an analytical rather than a critical review of Mr. Malcolmson's work. The subject treated at the greatest length, that of beriberi, being at least under the point of view in which the author presents it, comparatively new to the European reader, we feared that any direct commentary from us was calculated rather to obscure than illustrate the text; whilst the relation which this disease bears to the spinal affections observed at home, would make a more permanent and practical impression on the mind if discovered by the European practitioner, than if suggested by ourselves. We understand that copies of the work are very scarce, and so conscious are we of the impossibility of giving an adequate view of its valuable contents within the ordinary compass of an article in a journal, that we venture to suggest whether justice to the work, and to the object of its original publication, general utility, does not require that another edition should be published.

---

